# Identification and characterization of *PAL* genes involved in the regulation of stem development in *Saccharum spontaneum* *L*.

**DOI:** 10.1186/s12863-024-01219-9

**Published:** 2024-04-30

**Authors:** Xiaoqing Wu, Zetian Cui, Xinyi Li, Zehuai Yu, Pingping Lin, Li Xue, Abdullah Khan, Cailan Ou, Zuhu Deng, Muqing Zhang, Wei Yao, Fan Yu

**Affiliations:** 1 State Key Laboratory for Conservation and Utilization of Subtropical Agro-bioresources, Guangxi Key Laboratory for Sugarcane Biology, Academy of Sugarcane and Sugar Industry, Guangxi University, Nanning, 530004 China; 2 National Engineering Research Center for Sugarcane, Fujian Agriculture and Forestry University, Fuzhou, Fujian 350002 China; 3 Key Laboratory of Sugarcane Biology and Genetic Breeding, Ministry of Agriculture and Rural Affairs, Fujian Agriculture and Forestry University, Fuzhou, 350002 China

**Keywords:** Sugarcane, *Saccharum spontaneum*, Phenylalanine ammonia-lyase, Stem growth

## Abstract

**Background:**

*Saccharum spontaneum* *L*. is a closely related species of sugarcane and has become an important genetic component of modern sugarcane cultivars. Stem development is one of the important factors for affecting the yield, while the molecular mechanism of stem development remains poorly understanding in *S. spontaneum*. Phenylalanine ammonia-lyase (*PAL*) is a vital component of both primary and secondary metabolism, contributing significantly to plant growth, development and stress defense. However, the current knowledge about *PAL* genes in *S. spontaneum* is still limited. Thus, identification and characterization of the *PAL* genes by transcriptome analysis will provide a theoretical basis for further investigation of the function of *PAL* gene in sugarcane.

**Results:**

In this study, 42 of *PAL* genes were identified, including 26 *SsPAL* genes from *S. spontaneum*, 8 *ShPAL* genes from sugarcane cultivar R570, and 8 *SbPAL* genes from sorghum. Phylogenetic analysis showed that *SsPAL* genes were divided into three groups, potentially influenced by long-term natural selection. Notably, 20 *SsPAL* genes were existed on chromosomes 4 and 5, indicating that they are highly conserved in *S. spontaneum*. This conservation is likely a result of the prevalence of whole-genome replications within this gene family. The upstream sequence of *PAL* genes were found to contain conserved cis-acting elements such as G-box and SP1, GT1-motif and CAT-box, which collectively regulate the growth and development of *S. spontaneum*. Furthermore, quantitative reverse transcription polymerase chain reaction (qRT-PCR) analysis showed that *SsPAL* genes of stem had a significantly upregulated than that of leaves, suggesting that they may promote the stem growth and development, particularly in the + 6 stem (The sixth cane stalk from the top to down) during the growth stage.

**Conclusions:**

The results of this study revealed the molecular characteristics of *SsPAL* genes and indicated that they may play a vital role in stem growth and development of *S. spontaneum*. Altogether, our findings will promote the understanding of the molecular mechanism of *S. spontaneum* stem development, and also contribute to the sugarcane genetic improving.

**Supplementary Information:**

The online version contains supplementary material available at 10.1186/s12863-024-01219-9.

## Background

Sugarcane is an important economic crop worldwide, accounting for about 75 per cent of the world’s sugar production [1]. With its high sucrose accumulation and high tillering ability, making it become the highest tonnage crop worldwide [2]. Sucrose is produced in the leaves and stored in the cane stems through the phloem [3]. Thus, the growth and development of cane stem is the main factor affecting the yield of sugarcane. *Saccharum spontaneum L*. is a wild germplasm resource of *Saccharum* genus [4]. It is notable attributes such as high fiber content, lodging resistance, and disease resistance making it become an important genetic resource in sugarcane breeding [5]. Modern sugarcane cultivars were bred through the “noble” cross between *S. officinarum* and *S. spontaneum* [6]. Furthermore, these cultivars contain 10%∼20% of *S. spontaneum* lineages [7, 8]. Therefore, exploring these genes involved in stem development of *S. spontaneum* will be beneficial for sugarcane breeding.

Phenylalanine ammonia-lyase (*PAL*) is a key enzyme in the phenylalanine metabolic pathway of plants [9]. It plays a crucial role in plant growth, development and resistance to biotic and abiotic stresses [10–12]. *PAL* is encoded by a small multi-gene family with various members in different species [13–18]. It is the first rate-limiting enzyme in the phenylalanine metabolic pathway and catalyze the synthesis of natural substances such as lignin, flavonoids and anthocyanin [19–22]. Specifically, lignin is the main component of the secondary cell wall and is closely associated with stem morphology and secondary wall formation [23]. It is an essential component for providing mechanical support to plants and also transporting water, mineral and photosynthetic products. In addition, it also offers protection against pathogen invasion and promote plant growth and development [24–26]. Therefore, *PAL* plays a positive key role in the growth, development and survival of vascular plants. Flavonoids, as one of the phenolic compounds, not only act as unique UV filters protecting plants from UV radiation damage [27–28], but also has roles in anti-freezing, drought resistance, heat adaptation and frost resistance [29]. Anthocyanins serves as strong antioxidants in plant cells that assisting resist biotic and abiotic stresses, and attracting insects for pollination and seed dispersal [30–31]. Responding to various stresses, *PAL* can rapidly induce the expression of *PAL* genes at the transcriptome level, thus affecting the expression levels of *PAL* genes and protecting plants [32].

The *PAL* gene family plays an irreplaceable role in plant growth and development. As an important wild species, *S. spontaneum* has been widely used in sugarcane breeding. Although *PAL* genes have been studied in various plants, limited attention has been paid to the members of the PAL enzyme family and their expression patterns in *S. spontaneum*. Therefore, it is necessary to understand the evolutionary mechanism and expression pattern of *PAL* genes in *S. spontaneum*. This exploration will establish foundation for identifying gene function related to stem developmental mechanisms. In the present study, based on the transcriptomic data of AP85-441, we identified 26 *PAL* genes of *S. spontaneum* and further analyzed the physicochemical properties, sequence characteristics, phylogeny, gene structure, cis-regulatory element prediction and expression pattern of *PAL* gene family. This comprehensive analysis will provide insights into the biological functions of *PAL* gene and contribute to understand the stem development in *S. spontaneum*.

## Materials and methods

### Plant materials

The experimental material is *S. spontaneum* SES208 (2*n* = 8*x* = 64), which grown in the greenhouse of the Sugarcane Research Institute of Guangxi University. To verify the reliability of the download transcriptome, we collected leaves at maturity and stems at internodes + 3, +6 and + 9 of *S. spontaneum* respectively, and then total RNA was extracted for qRT-PCR experiments.

### Determination of *S. Spontaneum PAL* gene family members

To identify members of the *PAL* gene family, genomic data were collected for five species. Genomic data off *S. spontaneum* were downloaded from the published genome database (http://sugarcane.zhangjisenlab.cn/sgd/html/index.html) [33]. The haploid reference genome off R570 was obtained from the Sugarcane Genome Center (http://sugarcane-genome.cirad.fr/) [34]. Genomic data and protein sequences off sorghum, maize, and rice were downloaded from Phytozome (https://phytozome.jgi.doe.gov/) and EnsemblPlant (http://plants.ensembl.org/index.html) databases, respectively. The identification process involved several steps. Firstly, the Hidden Markov Model (HMM) search program [35] was used to search for protein sequences containing *PAL* structural domains. The HMM configuration file (PF00221) predicted by the Pfam database [36] was utilized for this purpose. Secondly, the protein sequences of four *Arabidopsis thaliana PAL* genes were downloaded from the *Arabidopsis* database (http://www.arabidopsis.org/), and using Blastp software. The *PAL* proteins in the three genomic databases were searched to identify candidate genes of the *PAL* family. Finally, the conserved domain of each candidate gene was further verified using the online tool NCBI CD-search (https://www.ncbi.nlm.nih.gov/Structure/cdd/wrpsb.cgi). Any genes with incomplete domain were excluded, resulting in the selection candidate genes for the *PAL* gene family. 

### Physicochemical properties and phylogenetic analysis of the *PAL* gene family

The physical and chemical properties of *PAL* family were predicted using the ExPasy online website (https://web.expasy.org/protparam). Protein sequences of *PAL* families from *Arabidopsis*, *Sorghum*, *S. spontaneum*, R570, maize, and rice were aligned using ClustalW multiplex sequence alignment with default parameters. The aim of this alignment was to investigate the phylogenetic relationships among *PAL* genes. The resulting alignment was then used to construct phylogenetic by the Neighbor-Joining (NJ) method of MEGA-X software. The calibration parameter bootstrap was set to 1000, while the remaining parameters were kept at their default values.

### Analysis of *PAL* gene family gene structure and conserved motifs

The CDS and gene sequences of both *PAL* family members were extracted from the *Sorghum* genome, R570 genome and *S. spontaneum* genome annotation files. The gene structures were obtained using the Gene Structure Display Server 2.0 (http://gsds.gao-lab.org/) online tool. To analyze the conserve motifs of PAL family protein sequences, the MEME Suite online tool (https://meme-suite.org/meme/tools/meme) [37] was employed with the number of searches was set to 10. Finally, the results obtained from the phylogenetic tree, conserved motifs analysis and gene structure analysis were integrated and visualized by using TBtools software.

### *PAL* gene family cis-acting element and covariance analysis

Based on the genome annotation information of *S. spontaneum*, *Sorghum* and R570, the CDS upstream 2000 bp transcription start site promoter sequence of each member of *PAL* gene family was extracted using the Gtf/Gff sequence extraction tool in TBtools software. The extracted sequences were then submitted to PlantCare [38] for cis-element prediction. This process is crucial for understanding and manipulating the regulatory mechanisms of *PAL* gene family members in *S. spontaneum*. Chromosomal location of *PAL* genes was obtained based on the genome annotation information. The covariance analysis software MCScanX was used to detect gene duplication events, intra- and inter-species covariance relationships in the *PAL* gene family [39], followed by chromosome localization [40]. Furthermore, the gene covariance analysis and visualization were conducted using the default parameters of TBtools software.

### Analysis of *SsPAL* gene expression pattern

The transcriptome expression profiles of *S. spontaneum* from various tissues, growth stages, developmental leaves, and day-night rhythms were downloaded from the public sugarcane genome database (http://sugarcane.zhangjisenlab.cn/sgd/html/index.html) [41]. The FPKM (Fragments Per Kilobase of exon model per Million mapped fragments) values of *SsPAL* genes were calculated using logarithmic function. Gene expression heatmaps were generated using TBtools software.

### Total RNA extraction and qRT-PCR analysis

Mature leaves of *S. spontaneum* and stem sections between the + 3, +6, and + 9 stems were collected and immediately frozen in liquid nitrogen for storage at -80 °C. Total RNA was extracted using TRIZOL reagent (Takara, Japan) and reverse transcribed into cDNA using PrimeScript™ RT reagent Kit with gDNA Eraser (Perfect Real Time) (Takara, Japan). qRT-PCR was performed using primers designed from the NCBI primer database. The LightCycler® 96 instrument (Roche, Switzerland) was used to compare relative expression differences between different stem sections and leaves, thereby validating transcriptome data. The relative expression levels were calculated using the 2-ΔΔCt method with 25 S rRNA of sugarcane as reference gene. The experiment included three biological replicates [42]. qRT-PCR reaction system consisted of 1 µL cDNA, 10 µL 2×ChamQ SYBR Master Mix, 2 µL of each forward and reverse primer (10 µmol/L), and 5 µL ddH_2_O. The reaction profile was as follows: 95 °C for 30s, followed by 40 cycles of 95 °C for 10s, 60 °C for 30s, and 95 °C for 10s. Then results were statistically analyzed (Student’s t-test) and the data were plotted using GraphPad Prism 8.0 software.The used primers are shown in Table S1.

## Results

### Identification of *PAL* gene family members in *S. bicolor*, *S. spontaneum*, and sugarcane cultivar R570

A total of 42 *PAL* genes were obtained from the *S. bicolor*, *S. spontaneum*, and R570 genome databases. Among these, eight genes were identified in sorghum, 26 in *S. spontaneum*, and eight in R570. These 42 genes were named according to their chromosomal positions on three genomes, and the allelic genes of *SsPAL* were designated as a, b, c, d, following previous naming conventions (Table 1). Results showed that *SsPAL4* and *SsPAL15* have 4 allelic genes, while most *SsPAL*s (15 genes) had no allele that indicated they have been lost during the evolutionary processes (Table 1). We then conducted the protein primary structure prediction on *PAL* genes, suggesting that its amino acid length was around 700 in sugarcane cultivar R570 and sorghum, with an isoelectric point ranging from 5.6 to 6.26, a molecular weight of 75.60-83.12 KD, protein instability coefficient ranging from 29.1 to 37.42, and average hydrophobicity ranging from − 0.138 to -0.02 (Table S2). These results implied that all *PAL* genes in sugarcane cultivars R570 and sorghum *PAL* gene families are stable acidic proteins. In *S. spontaneum*, the amino acid length of most *PAL* genes was around 700, while *SsPAL*5a and *SsPAL*16 were 1054 and 1693, respectively (Table S2). Except for *SsPAL*5a, the isoelectric points of other family members are between 5.69 and 7.56. Most of the molecular weights are below 100 KD, while *SsPAL*1 and *SsPAL*2 have molecular weights greater than 100 KD. Except for the protein instability coefficients *SsPAL*1 was 29.65, the remaining genes belong to stable acidic proteins (Table S2).

**Table 1:** The members of *PAL* gene family in *S. spontaneum*

Gene Name	Gene ID	Copy Type
*SsPAL1*	Sspon.04G0008040-9P	1
*SsPAL2*	Sspon.04G0008040-2P	4
*SsPAL3*	Sspon.04G0008040-1T	4
*SsPAL4a*	Sspon.04G0008040-1 A	3
*SsPAL4b*	Sspon.04G0008040-2B	4
*SsPAL4c*	Sspon.04G0008040-3 C	4
*SsPAL4d*	Sspon.04G0008040-4D	4
*SsPAL5a*	Sspon.04G0008060-1 A	4
*SsPAL5b*	Sspon.04G0008060-2B	4
*SsPAL5c*	Sspon.04G0008060-3 C	2
*SsPAL6*	Sspon.04G0008040-1P	4
*SsPAL7*	Sspon.04G0008040-3P	3
*SsPAL8*	Sspon.04G0024420-1B	4
*SsPAL9*	Sspon.04G0032220-1 C	4
*SsPAL10*	Sspon.04G0008040-6P	4
*SsPAL11*	Sspon.04G0008070-3 C	4
*SsPAL12*	Sspon.04G0008040-11P	4
*SsPAL13*	Sspon.04G0008040-8P	4
*SsPAL14*	Sspon.04G0008040-4P	4
*SsPAL15a*	Sspon.05G0007010-1 A	4
*SsPAL15b*	Sspon.05G0007010-2B	4
*SsPAL15c*	Sspon.05G0007010-3 C	4
*SsPAL15d*	Sspon.05G0007010-4D	4
*SsPAL16*	Sspon.04G0008040-5P	4
*SsPAL17*	Sspon.04G0008040-7P	4
*SsPAL18*	Sspon.04G0008040-10P	4

*Note* The gene replication types are denoted as: 1: dispersed duplication; 2: proximal duplication; 3: tandem duplication; 4: the whole-genome duplication or segmental duplication

### Construction of a phylogenetic tree of *PAL* gene family in *S. spontaneum*

**Fig. 1:**
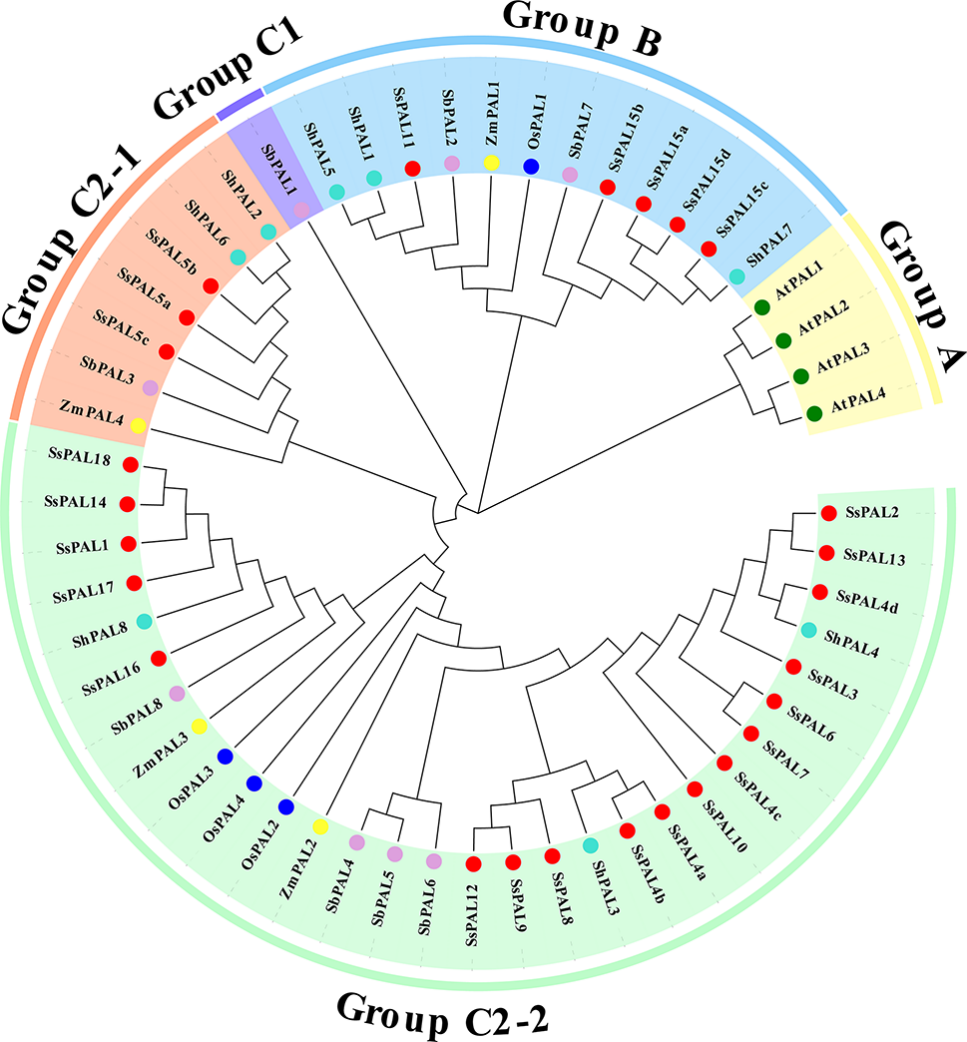
Phylogenetic tree of *PAL* genes family among different species. *Ss: S. spontaneum, At: A. tricolor, Os: O. sativa, Zm: Z. mays, Sb: S. bicolor, Sh*: sugarcane cultivar *R570.* The different colors indicated different groups of *PAL genes* in various species

To explore the evolutionary relationship of *PAL* family members, we screened the *PAL* protein sequences of *Z. mays*, *O. sativa*, *S. spontaneum*, *S. bicolor*, sugarcane cultivar R570 and *A. tricolor*. Then, a phylogenetic tree was constructed using MEGA-X. Phylogenetic analysis indicated that *PAL*s of different plants could be divided into 3 groups, Group A, Group B, and Group C (Fig. 1). Of these, Group C further divided into two subgroups, C1 and C2-1/2 branches. Group C1 has one *SbPAL1,* suggesting that this gene had diverged from other genes in *S. bicolor* (Fig. 1). Group A only contained *AtPALs,* indicating that the significant differences of *PAL*s between monocotyledonous and dicotyledonous (Fig. 1). *SsPAL* genes were unevenly distributed among Group B and Group C2-1/2, where Group C2-2 contained the highest number of *SsPAL* family members (contained 18 *SsPAL* genes). Furthermore, these results showed that *SsPALs* were closest to *ShPALs*, followed by *SbPALs*, *ZmPALs* and Os*PALs* (Fig. 1).

### Analysis of the gene structure and conserved structural domains of *S. spontaneum PAL* gene family

**Fig. 2:**
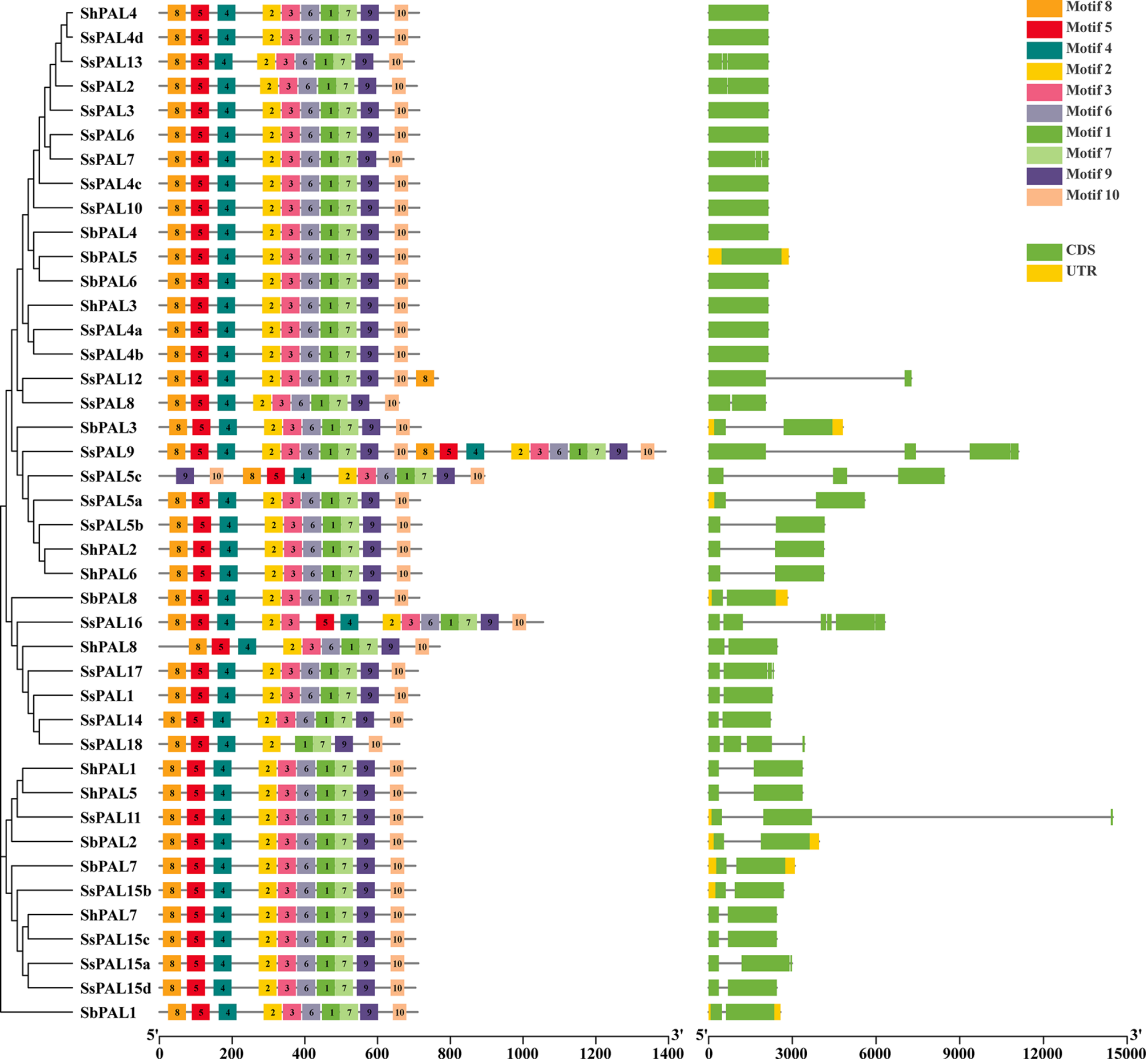
Analysis of conserved motifs and gene structure of *PAL* gene family in *S. spontaneum.* The different colored modules represent different motifs of *PAL* genes. CDS stands for exon. UTR stands for intron

Conservative gene structures may provide insights to the key events in the evolution of genes. We predicted the exon-intron and conserved motifs of *SsPAL*, *SbPAL* and *ShPAL* genes. The conserved motif composition and number analysis of the genes were done by using MEME software, and 10 conserved motifs were identified (named motif 1–10) (Fig. 2). Results showed that all *PAL* proteins contained motif 1–10 in these three species. In most proteins, motif 4, 5 and 8 are always closely linked to motif 1, 2, 3, 6, 7, 9 and 10, indicating that they were conserved in the majority of members (Fig. 2). All *PAL*s contained exons, mostly have 1 to 2, while a few have 3 or 5 exons. In contrast, introns of *PAL* gene family members are more stable. *ShPAL* family does not contain introns, while *SbPAL1*, *SbPAL2*, *SbPAL3*, *SbPAL5*, *SbPAL7*, *SbPAL8*, *SsPAL5a*, *SsPAL11* and *SsPAL15b* have 1-2 introns. This indicated that the structural differences among members of *SbPAL* gene family are not significant, and only a few members exhibit variation in intron-exon organization. The original gene structures are less complex than those of *SsPAL* gene family.

### Analysis of promoter cis-acting elements

**Fig. 3:**
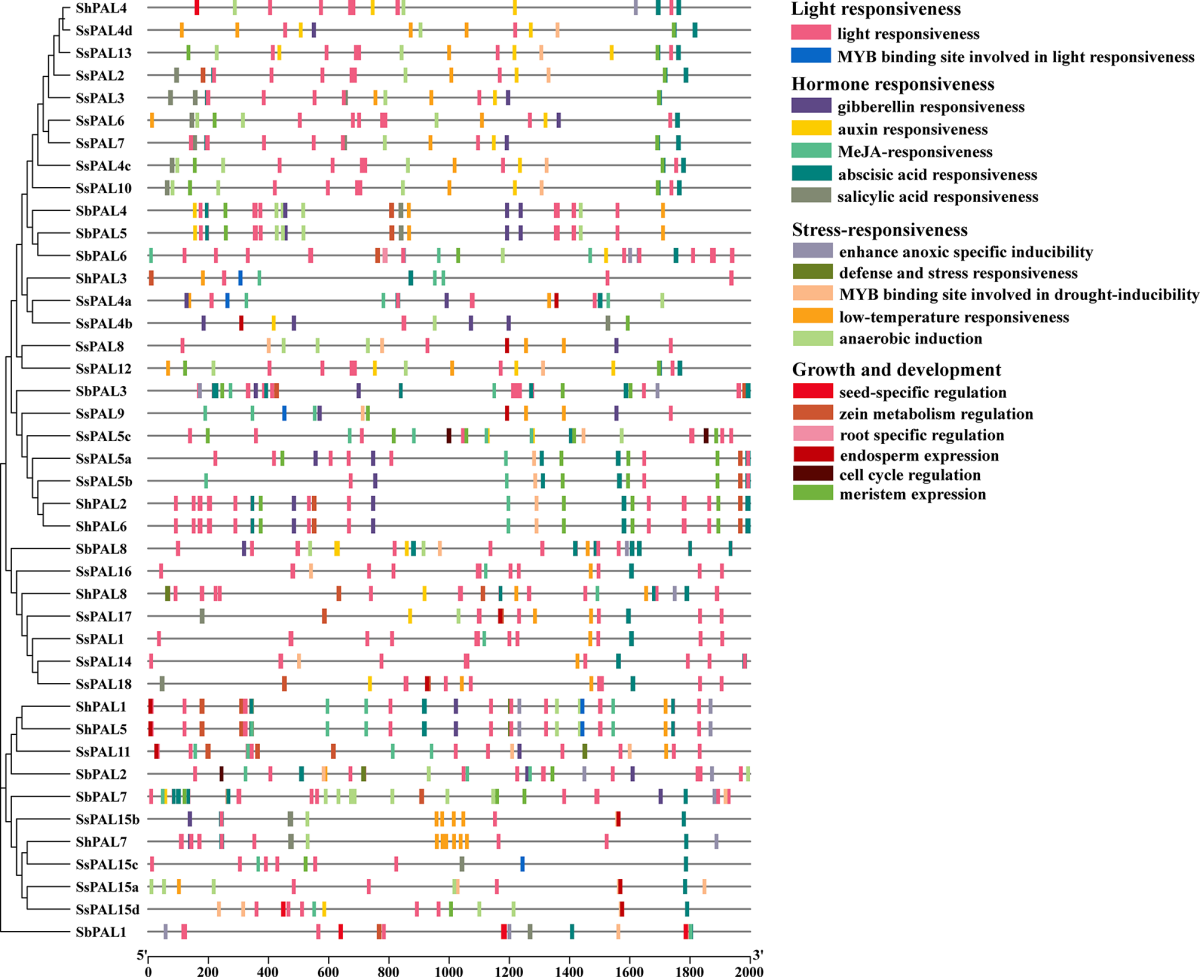
Distribution of cis-acting elements of the *PAL* family in *S. spontaneum.* Cis-elements with similar functions are displayed in the same color boxes. The grey line indicates the promoter region length of the *PAL* genes

**Fig. 4:**
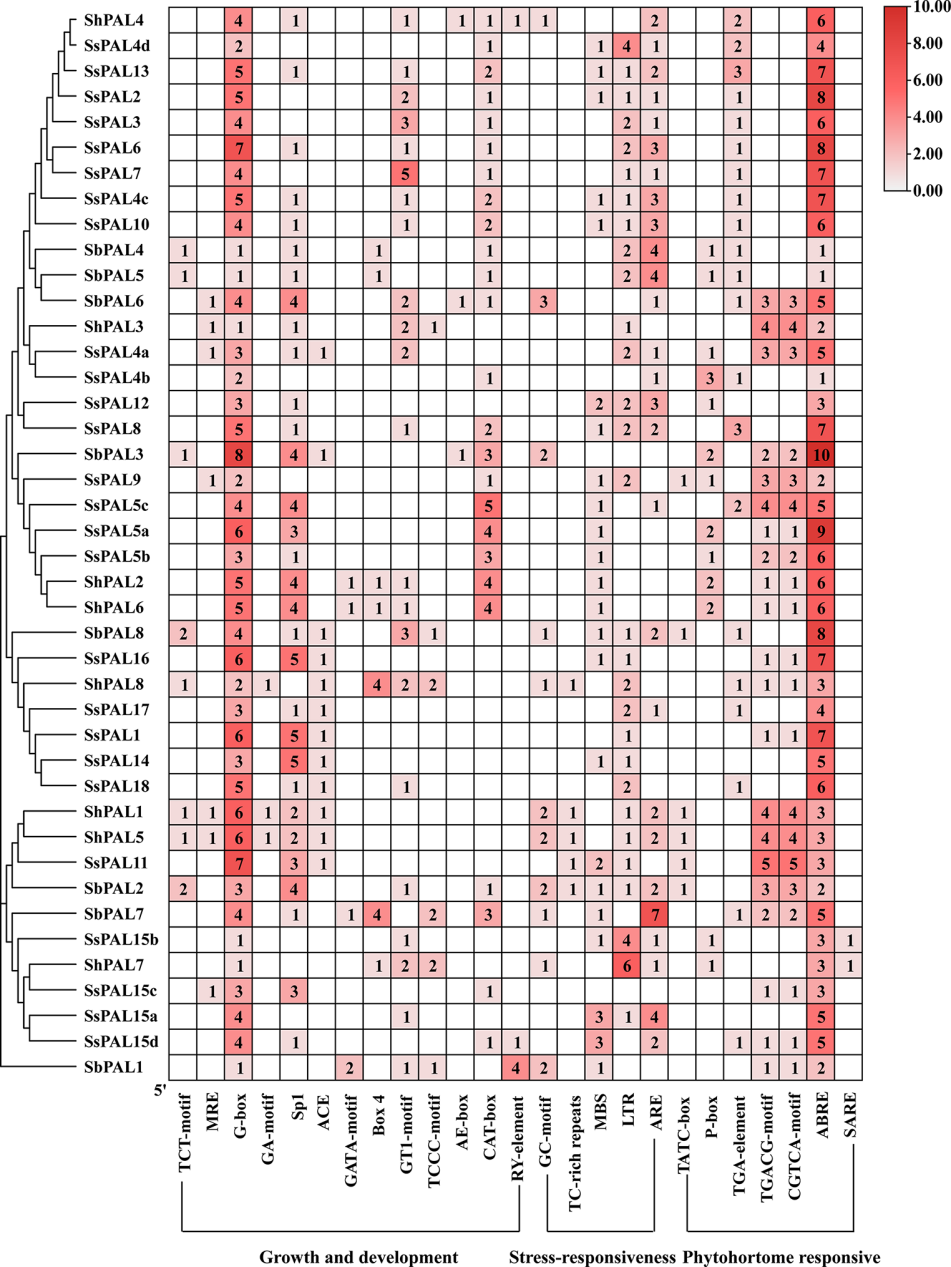
Heat map of the distribution and number of cis-acting elements of the *PAL* promoter in *S. spontaneum*. The number of cis-elements from the *PAL* genes were shown in heatmap boxes. Blank box means no corresponding cis-elements

The cis-acting elements in the promoter region play a critical role in controlling gene transcription and expression. Analyzing promoter cis-acting elements can improve the understanding of gene function. In this study, we predicted 2,000 bp sequences upstream of *PAL* gene family members using the PlantCARE online website, and a total of 18 cis-acting elements were identified (Fig. 3). These different cis-acting elements could be functionally classified into four major categories: light-responsive elements, hormone-responsive elements, stress-responsive elements and plant growth metabolism-responsive elements. The analysis of cis-acting element showed that the promoter motifs of *S. spontaneum PAL* gene are involved in a variety of biological processes. All *SsPAL* genes contained light-responsive elements, with the conserved G-Box being the most prevalent element (100%), followed by sp1 (69.2%), meristematic tissue expression element (CAT-box) (61.5%) and GT1-motif (46.2%) (Fig. 4). Among the hormone response elements, abscisic acid response elements (ABRE) involved in abscisic acid response were found abundance across the three gene families. With some genes containing a high number of ABRE elements, such as *SbPAL3* was up to 10 elements. Among the stress response elements, low temperature response elements (LTR) were relatively abundant, suggesting their potential involvement in stress regulation. These results indicated that *SsPAL* genes are not only widely involved in the growth and development of sugarcane, but also regulate various stress responses.

### Collinearity analysis reveals pervasive gene duplications

**Fig. 5:**
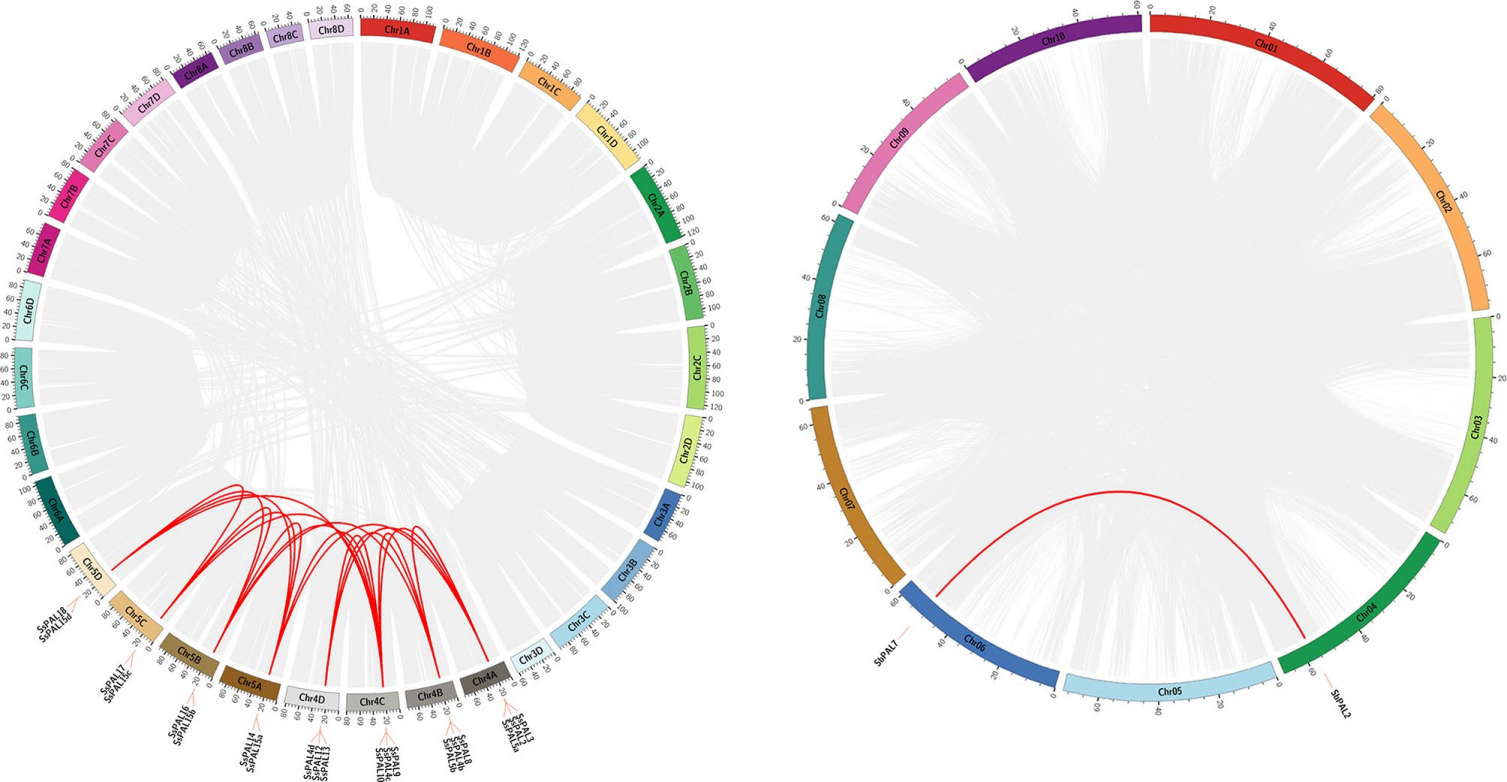
Distribution and collinearity analysis of *SsPAL* genes in *S. spontaneum* and *Sorghum bicolor*. The inner line indicates the covariance within the *SsPAL* and *SbPAL* genes

To further infer the phylogenetic mechanism of the *PAL* gene family, the collinearity of *SsPAL* gene family was analyzed using McScanX software. Results showed that 34 pairs of homologous genes were existing among members of *SsPAL* gene family, including 26 pairs occurred in alleles and 8 pairs occurred in non-alleles, and 26 pairs (76.47%) within homologous chromosome groups (Fig. 5, Table S3). It was found that whole genome replication or segmental replication (84.62%) was the main replication type of *SsPAL* genes, which was the main way of *SsPAL* gene family expansion that accord with the characteristics of gene family expansion in polyploid species (Table S3). Therefore, the mainly reason for the significantly higher number of *SsPAL* genes family than other crops such as *Arabidopsis* and rice is the polyploid nature of *S. spontaneum* resulting from the whole genome duplication and chromosome doubling events. Similarly, collinearity analysis of *SbPAL* genes family revealed that only one pair of homologous genes were distributed in chromosomes4 and 6, respectively. These non-allelic genes may have originated from segmental duplication events.

### Analysis of the spatio-temporal expression pattern of *SsPAL* genes

**Fig. 6:**
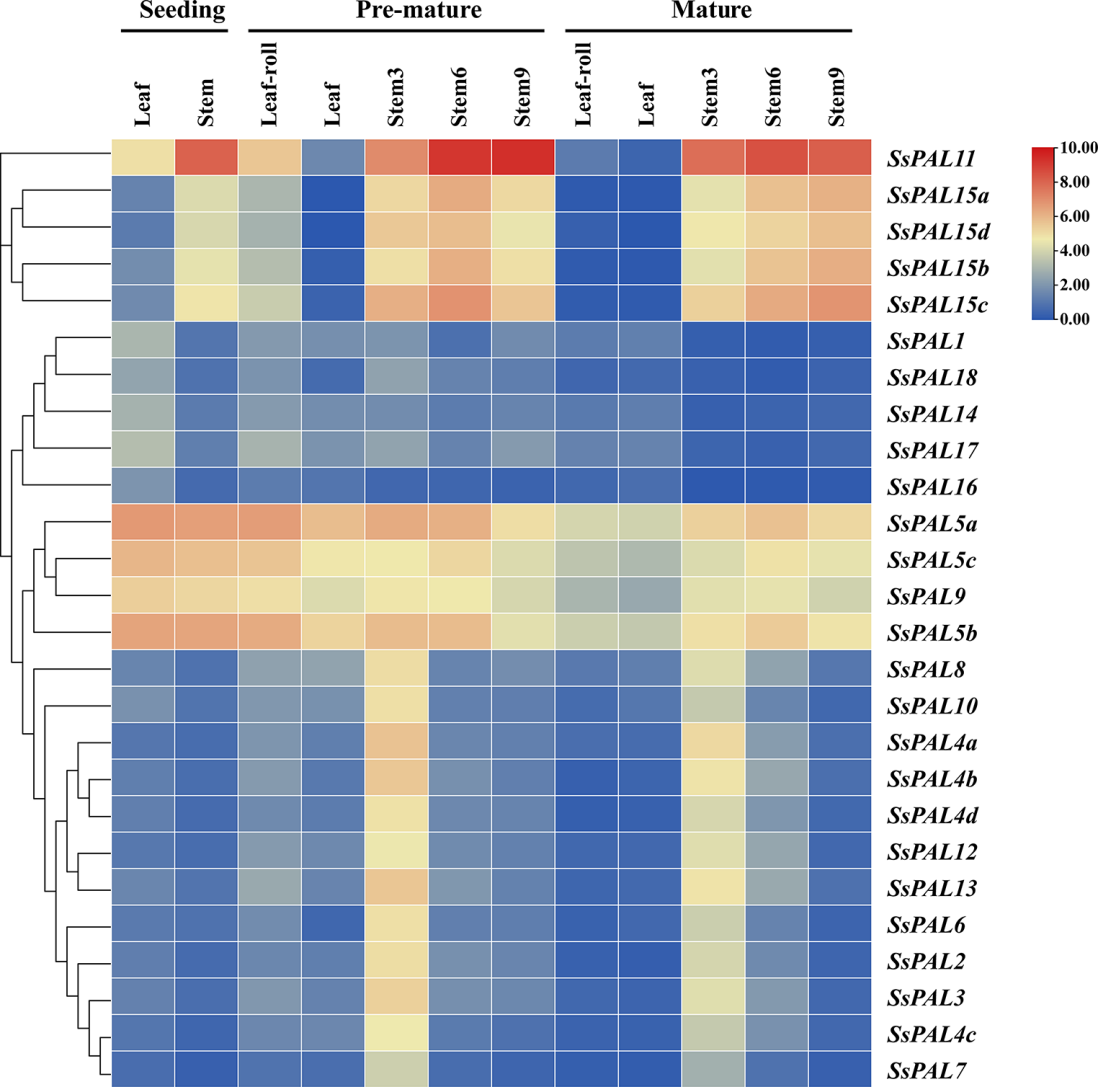
Expression pattern of *SsPAL* gene in different period and stages of *S. spontaneum.* Seeding refers to seedling stage, Pre-mature refers to the elongating stage, Mature refers to maturity stage

The temporal and spatio-temporal expression pattern of *PAL* genes in *S. spontaneum* were investigated using transcriptomic data from different tissues and leaf developmental gradients. Five genes (*SsPAL1, SsPAL18, SsPAL14, SsPAL17 and SsPAL16*) exhibited low or no expression in both stem and leaves across the developmental stages of *S. spontaneum* (Fig. 6). In addition to these 5 genes, most other *SsPAL* genes showed higher expression in the stem than that of the leaves, indicating their important role in the growth and elongation of stalks. The expression leaves of + 6 stems were more significant compared to + 9 and + 3 stems, indicating higher expression during both pre-mature and mature stages. This suggests that these genes may have a stronger biosynthetic function during the vigorous growth stage, contributing to the synthesis of compounds such as lignin.

**Fig. 7:**
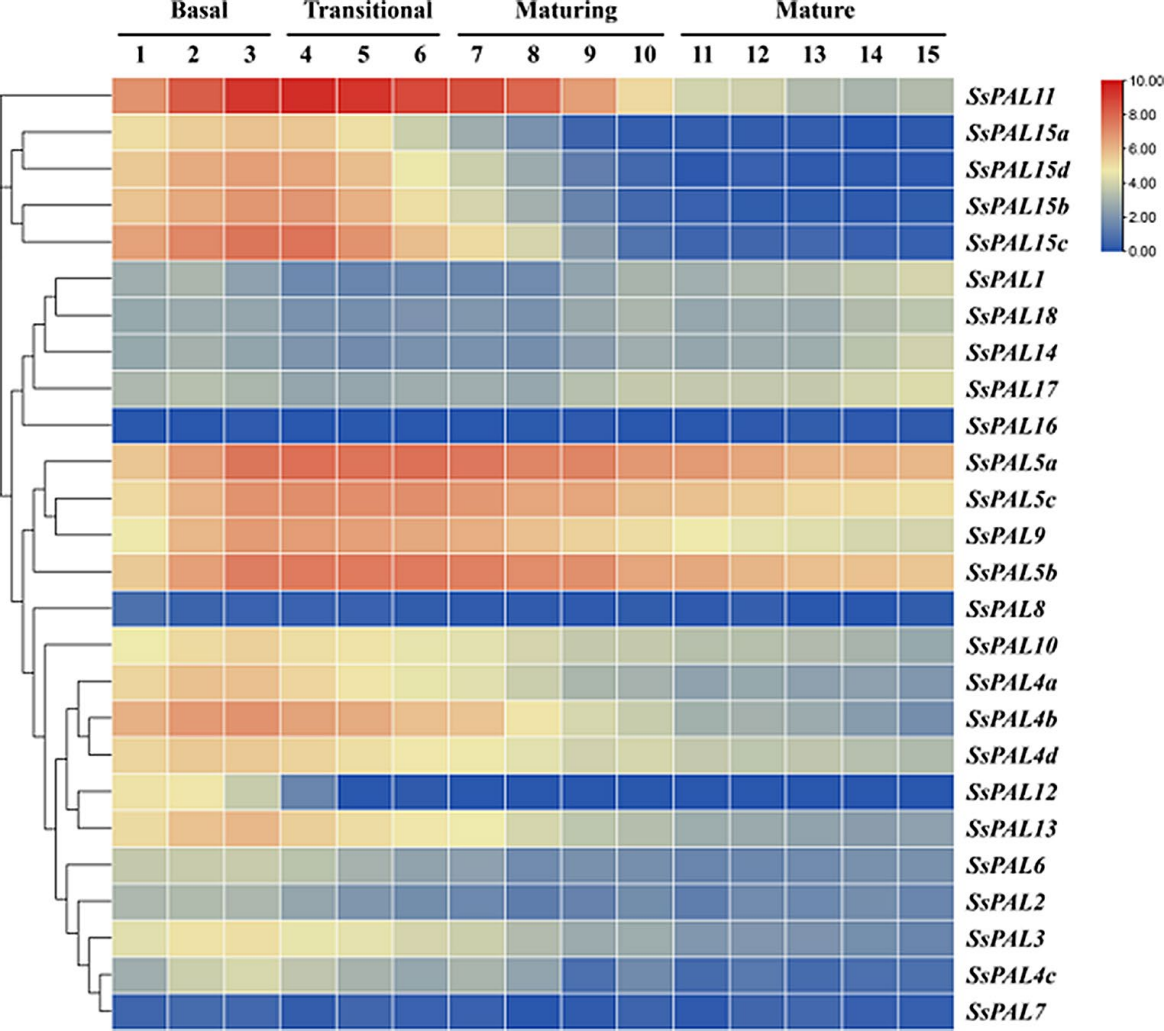
Expression of *SsPAL* gene in different leaf development gradients of *S. spontaneum L.* 1 ∼ 15 refers to different locations of leaf segment parts around 1 cm length. Basal refers to leaf base. Transitional refers to source bank transition region. Maturing refers to mid maturity. Mature refers to maturity stage

To elucidate the functional differentiation of *S. spontaneum PAL* gene family in photosynthetic tissues, expression analysis was performed on a continuous gradient model of *S. spontaneum* development. *SsPAL16*, *SsPAL18* and *SsPAL7* were not expressed in leaves with different developmental gradients, indicating their very limited role in leaf development of *S. spontaneum*. Most other *SsPAL* genes exhibited a relatively high level of expression in the base, transition zone, and the first half of mature zone 1 of leaves (Fig. 7). High level of expression at the base of the leaf may be due to its role as a source pool transition zone, which is closer to the stalk and contains higher lignin content compared to other parts. The above results suggest that these genes also have an effect on the growth of specific regions during the leaf development.

### Validation of relative expression levels of *SsPAL* gene family by qRT-PCR

**Fig. 8:**
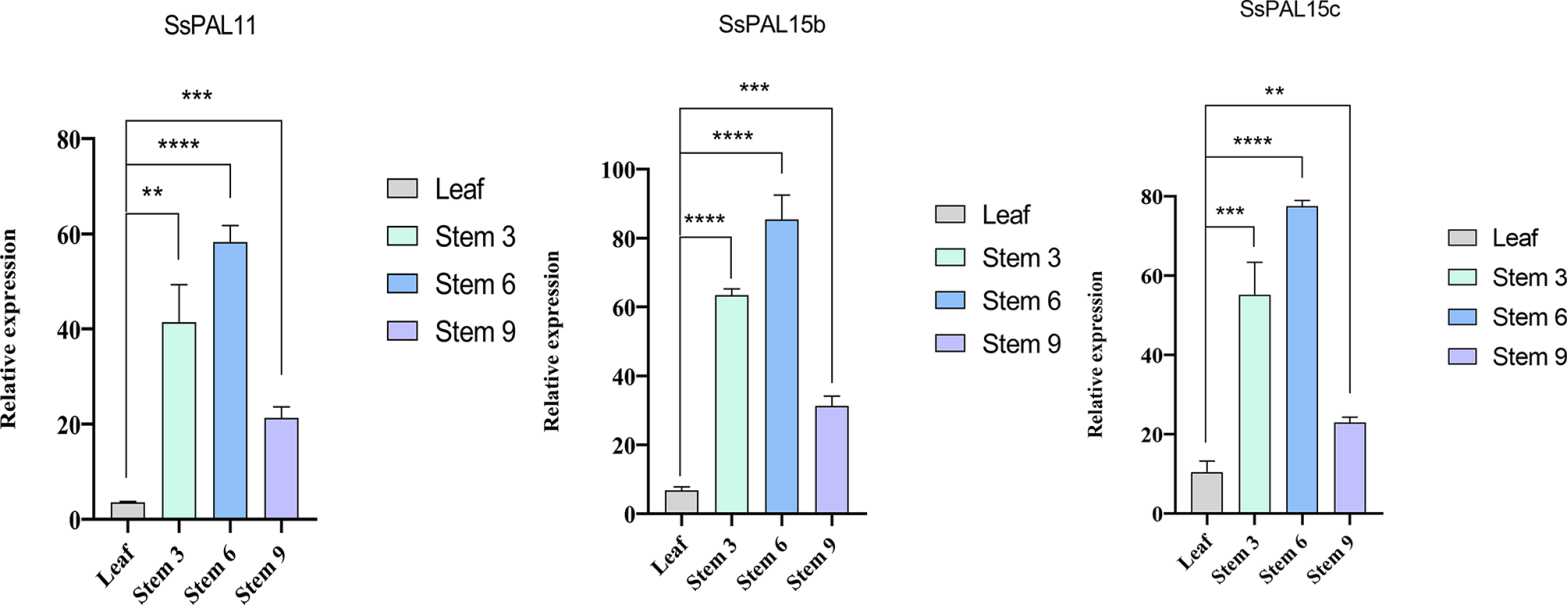
qRT-PCR validation of the relative expression of *SsPAL* gene in *S. spontaneum * leaves and stems. Leave: *S. spontaneum* leaves at maturity; Stem 3: the 3rd stem node of *S. spontaneum* at maturity; Stem 6: the 6th stem node of *S. spontaneum* at maturity; Stem 9: the 9th stem node of *S. spontaneum* at maturity

In order to compare the expression levels between different tissues of *S. spontaneum* and to validate the results of the expression pattern analysis described above. We chose the first leaf and the +3, +6 and +9 stems of *S. spontaneum* as the experimental materials. Three *SsPAL* genes were randomly selected for qRT-PCR validation. Results of gene expression showed that in the stems and leaves of mature *S. spontaneum* SES208, the expression levels of these three genes in the stem sections between the +3, +6, and +9 stem were higher than that of the leaves. In particularly, the mean expression levels of *SsPAL* gene in + 3, +6 and + 9 stems were about 7.72, 10.68 and 3.65 times higher than that of + 1 leaves, respectively. This result suggests that the *SsPAL* gene plays an important role in stem development. The differences in expression levels were found to be extremely significant, which is consistent with the expression pattern in the transcriptome data. Moreover, all three genes exhibited higher expression levels in the + 6 stem compared to + 3 stem, which aligns with the previous hypothesis (Fig. 8).

## Discussion

Phenylalanine ammonia-lyase (*PAL*) plays an important role in plants as a link between primary and secondary metabolism. As a key enzyme in the metabolic pathway of phenylpropane, *PAL* plays an important role in plant growth, development and resistance in a widely species [11–12, 43–46]. The yielding of *S. spontaneum* depends on the growth and development of its stems. However, there are few studies on the *PAL* gene family of *S. spontaneum* involved in stem growth and development. Therefore, exploring the molecular characteristics of *PAL* genes in *S. spontaneum* is important for further improving the sugarcane agronomic trait. *PAL* is a conserved multigene family, while contains various gene number in different species. For example, *Arabidopsis* [47] and tobacco [48] both contain 4 *PALs*, sorghum [49] contains 8 *PALs*, potato [16] contains 14 *PALs*, rice [50] contains 9 *PALs*, and cucumber [51] contains 13 *PALs*. In this study, we identified 26 *S. spontaneum PAL* genes, suggesting the number of *S. spontaneum PAL* genes was significantly higher than that of rice, *Arabidopsis* and maize [52], but lower than that of wheat. This indicates that the number of *PAL* genes is stochastic among species, which is consistent with the results of previous studies [53].

In contrast to the results of previous studies, *S. spontaneum PAL* genes were distributed on only two chromosomes, and in combination with the type of replication of this gene family. Whole-genome replication led to a more conservative evolutionary and expansion of *S. spontaneum PAL* gene family, which resulting in a restricted distribution region. It was also one of the main reasons for the larger number of *S. spontaneum PAL* genes than that of *Arabidopsis* and other crops. Previous studies have shown a link between whole-genome duplication and plant morphological evolution. Whole-genome duplication can break the limits of purifying selection on gene evolution and allow genes to assume new functions [54]. Therefore, the genome-wide replication type of the *PAL* gene in *S. spontaneum* may be able to play a role in sugarcane breeding, such as thecontrol offlowering time, alteration of stem length and stem diameter size, and thus the variation of its sugar content.

The obvious difference between the genes of monocotyledonous and dicotyledonous plants resulted in *S. spontaneum PAL* gene being more closely homologous to graminaceous plants and more distantly homologous to *Amaranth*. *PAL*, the first key enzyme of the phenylpropane metabolic pathway, has maintained stability and convergence during genetic evolution. This is consistent with the results of previous studies [55, 56]. Except for *SsPAL18*, all the *PALs* proteins identified in this study have complete conserved structural domains and exhibit very similar alignments. However, there are also some unique motifs such as *SsPAL9*, *SsPAL5c* and *SsPAL16*, indicating the entire conserved structural domain facilitates the functional diversity of *SsPAL* genes in addition to performing the conserved biological functions of *S. spontaneum PAL* family members [47]. *PAL* is considered to be a master regulator of various abiotic stress responses and is involved in plant growth and development. The results of this study showed that the conserved light element G-box (100%), which is correspondingly associated with light, as well as sp1 (69.2%), GT1-motif (46.2%) and CAT-box (61.5%) are widely present in the upstream sequences of genes. This suggests that these genes regulate plant seed growth and meristematic tissue development, thus affecting plant growth metabolism, which is consistent with the above findings. These functional differences confirm that *PAL* is a multifunctional gene family.

The growth and development of stem is a critical factor affecting the yields of *S. spontaneum*. Our qRT-PCR results revealed that the expression of *PAL* gene was higher in stems than that of leaves, which was similar to the expression pattern of other plants [50, 57–58]. The expression of *PAL* genes in + 6 stem was higher than that of  + 3 and + 9 stems, which may be due to the fact that the growth and development of *S. spontaneum* is mainly depended on internode elongation. In the tissue closest to the *S. spontaneum* stalk, *SsPAL* was highly expressed in the base of the leaf with more lignin content than the other leaf parts. This suggests that *PAL* is controlled by a family of genes with different expression properties in different tissues and involved in different metabolic pathways [59]. The results of this experiment not only proved the reliability of the transcriptome data analysis of *S. spontaneum*, but also indicated that the *SsPAL* gene family might play an important function in stem development.

## Conclusions

In this study, a total of 26 *SsPAL* family members were identified in *S. spontaneum*, along with 8 *SbPAL* family members in sorghum and 8 *ShPAL* family members in modern sugarcane cultivars. Analysis of the physicochemical properties, gene structure, protein conserved structural domains, phylogeny, collinearity, and expression heat map of these members revealed that the *S. spontaneum PAL* family genes likely play a critical role in plant growth and development, especially in stem nodes. The findings suggest that these *SsPAL* genes could serves as potential genetic resources for sugarcane breeding, and provide basic information for further studies on the biological functions of *SsPAL* and promoting breeding efforts to enhance important traits in sugarcane.

### Electronic supplementary material

Below is the link to the electronic supplementary material.

Supplementary Material 1

## Data Availability

Data is provided within the manuscript or supplementary information files.

## Abbreviations

PAL: Phenylalanine ammonia-lyase
ABRE: Abscisic acid responsiveness element
qRT-PCR: Quantitative reverse transcription polymerase chain reaction
HMM: Hidden Markov Model
NJ: Neighbor-Joining

## References

[1] Parameswari B, Nithya K, Kumar S et al. Genome wide association studies in sugarcane host pathogen system for disease resistance: an update on the current status of research[J]. Indian Phytopathol, 2021(5).

[2] Dal-Bianco M, Carneiro MS, Hotta CT, Chapola RG, Hoffmann HP (2012). Sugarcane improvement: how far can we go?. Curr Opin Biotechnol.

[3] Moore PH (1995). Temporal and spatial regulation of sucrose accumulation in the sugarcane stem. Funct Plant Biol.

[4] Zhang Q, Qi Y, Pan H, Tang H, Wang G, Hua X (2022). Genomic insights into the recent chromosome reduction of autopolyploid sugarcane *Saccharum spontaneum*. Nat Genet.

[5] da Silva JA (2017). The importance of the Wild Cane *Saccharum spontaneum* for Bioenergy genetic breeding. Sugar Tech.

[6] Yu F, Wang P, Li X, Huang Y, Wang Q (2018). Characterization of chromosome composition of sugarcane in nobilization by using genomic in situ hybridization. Mol Cytogenet.

[7] D’Hont A, Grivet L, Feldmann P, Glaszmann JC, Rao S, Berding N (1996). Characterisation of the double genome structure of modern sugarcane cultivars (*Saccharum spp*) by molecular cytogenetics. Mol Gen Genet MGG.

[8] Piperidis G, Piperidis N, D’Hont A (2010). Molecular cytogenetic investigation of chromosome composition and transmission in sugarcane. Mol Genet Genomics.

[9] Feng YT, Huang QL, Zhang R (2022). Molecular characterisation of *PAL* gene family reveals their role in abiotic stress response in lucerne (Medicago sativa). Crop Pasture Sci.

[10] Vogt T (2010). Phenylpropanoid Biosynthesis. Mol Plant.

[11] Tonnessen, Bradley W (2015). Rice phenylalanine ammonia-lyase gene *OsPAL4* is associated with broad spectrum disease resistance. Plant Mol Biol.

[12] Xiao-Zhang Y, Wei-Jia F,,Yu-Juan L et al. Differential expression of the PAL gene family in rice seedlings exposed to chromium by microarray analysis.[J].Ecotoxicology (London, England),2018,27(3):325–335. 10.1007/s10646-018-1897-5 29404866

[13] Dehghan S, Sadeghi M, Pöppel A. Differential inductions of phenylalanine ammonia-lyase and chalcone synthase during wounding, salicylic acid treatment, and salinity stress in safflower, Carthamus tinctorius. Biosci Rep 2014, 34(3). 10.1042/BSR20140026 PMC4069684 24865400

[14] Raes J, Rohde A, Christensen JH (2003). Genome-wide characterization of the Lignification Toolbox in Arabidopsis. Plant Physiol.

[15] Reichert Angelika I, He X-Z, Dixon Richard A (2009). Phenylalanine ammonia-lyase (PAL) from tobacco (*Nicotiana tabacum*): characterization of the four tobacco *PAL* genes and active heterotetrameric enzymes1. Biochem J.

[16] Tonnessen BW, Manosalva P, Lang JM (2015). Rice phenylalanine ammonia-lyase gene *OsPAL4* is associated with broad spectrum disease resistance. Plant Mol Biol.

[17] Yan F, Li H, Zhao P (2019). Genome-wide identification and transcriptional expression of the PAL Gene Family in Common Walnut (Juglans Regia L). Genes.

[18] Mo F, Li L, Zhang C (2022). Genome-wide analysis and expression profiling of the phenylalanine Ammonia-lyase Gene Family in Solanum tuberosum. Int J Mol Sci.

[19] Li G, Wang H, Cheng X (2019). Comparative genomic analysis of the PAL genes in five Rosaceae species and functional identification of Chinese white pear. PeerJ.

[20] Chen YP, Li FJ, Tian L (2017). The phenylalanine Ammonia Lyase Gene LjPAL1 is involved in plant defense responses to Pathogens and Plays Diverse roles in Lotus japonicus-Rhizobium Symbioses. Mol Plant Microbe Interact.

[21] Bagal UR, Leebens-Mack JH, Lorenz WW. The phenylalanine ammonia lyase (PAL) gene family shows a gymnosperm-specific lineage. BMC Genomics 2012, 13. 10.1186/1471-2164-13-S3-S1 PMC3394424 22759610

[22] He J, Liu YQ, Yuan DY (2020). An R2R3 MYB transcription factor confers brown planthopper resistance by regulating the phenylalanine ammonia-lyase pathway in rice. Proc Natl Acad Sci USA.

[23] Yuan Y, Yang X, Feng M (2021). Genome-wide analysis of R2R3-MYB transcription factors family in the autopolyploid *Saccharum spontaneum*: an exploration of dominance expression and stress response. BMC Genomics.

[24] Qin Y, Li QE, An QJ (2022). A phenylalanine ammonia lyase from Fritillaria unibracteata promotes drought tolerance by regulating lignin biosynthesis and SA signaling pathway. Int J Biol Macromol.

[25] Gho YS, Kim SJ, Jung KH (2020). Phenylalanine ammonia-lyase family is closely associated with response to phosphate deficiency in rice. Genes Genomics.

[26] Zhao SS, Zhao L, Liu FX (2016). NARROW AND ROLLED LEAF 2 regulates leaf shape, male fertility, and seed size in rice. J Integr Plant Biol.

[27] Huang J, Gu M, Lai Z (2010). Functional analysis of the Arabidopsis PAL gene family in plant growth, development, and response to environmental stress. Plant Physiol.

[28] Pourcel L, Routaboul JM, Cheynier V (2007). Flavonoid oxidation in plants: from biochemical properties to physiological functions. Trends Plant Sci.

[29] Panche AN, Diwan AD, Chandra SR (2016). Flavonoids: an overview. J Nutritional Sci.

[30] Santos-Buelga C, Mateus N, Freitas D. Anthocyanins. Plant pigments and beyond. In., vol. 62. Journal of agricultural food chemistry: ACS Publications; 2014: 6879–6884. 10.1021/jf501950s 24970106

[31] Zhang ZC, Sun CQ, Yao YM (2019). Red anthocyanins contents and the relationships with phenylalanine ammonia lyase (PAL) activity, soluble sugar and chlorophyll contents in carmine radish (*Raphanus sativus* L). Hortic Sci.

[32] Ritter H, Schulz GE (2004). Structural basis for the entrance into the phenylpropanoid metabolism catalyzed by phenylalanine ammonia-lyase. Plant Cell.

[33] Zhang J, Zhang X, Tang H (2018). Allele-defined genome of the autopolyploid sugarcane Saccharum spontaneum L[J]. Nat Genet.

[34] Garsmeur O, Droc G, Antonise R (2018). A mosaic monoploid reference sequence for the highly complex genome of sugarcane. Nat Commun.

[35] Wheeler TJ, Eddy SR (2013). Nhmmer: DNA homology search with profile HMMs. Bioinformatics.

[36] Finn RD, Coggill P, Eberhardt RY (2016). The pfam protein families database: towards a more sustainable future. Nucleic Acids Res.

[37] Bailey TL, Johnson J, Grant CE (2015). The MEME suite. Nucleic Acids Res.

[38] Lescot M, Déhais P, Thijs G (2002). PlantCARE, a database of plant cis-acting regulatory elements and a portal to tools for in silico analysis of promoter sequences. Nucleic Acids Res.

[39] Wang Y, Tang H, Debarry JD (2012). MCScanX: a toolkit for detection and evolutionary analysis of gene synteny and collinearity. Nucleic Acids Res.

[40] Chen C, Chen H, Zhang Y (2020). TBtools: an integrative toolkit developed for interactive analyses of big biological data. Mol Plant.

[41] Li Z, Hua X, Zhong W (2020). Genome-wide identification and expression profile analysis of WRKY family genes in the autopolyploid Saccharum spontaneum. Plant Cell Physiol.

[42] Ling H, Wu Q, Guo J, Xu L, Que Y (2014). Comprehensive Selection of reference genes for gene expression normalization in sugarcane by Real Time quantitative RT-PCR. PLoS ONE.

[43] SONG X P, HUANG X, MO F L et al. Cloning and expression analysis of sugarcane phenylalanin ammonia-lyase(PAL) gene [J]. Scientia Agricultura Sinica, 2013, 46(14): 2856–2868.

[44] Li Y, Xihe Z, Kai Y (2019). Physiological mechanism of different varieties and potassium application amount on cotton resistance to Verticillium wilt[J]. Cotton Sci.

[45] Valcarcel J (2016). Levels of potential bioactive compounds including carotenoids, vitamin C and phenolic compounds, and expression of their cognate biosynthetic genes vary significantly in different varieties of potato (Solanum tuberosum L.) grown under uniform cultural conditions. J Sci food Agric vol.

[46] Xuejin Chen B. A, Identification of PAL genes related to anthocyanin synthesis in tea plants and its correlation with anthocyanin content. Hortic Plant J 8. 3(2022):381–94.

[47] Huang J et al. Functional analysis of the ArabidopsisPALGene Family in Plant Growth, Development, and response to environmental stress. Plant Physiol 153.4(2010):1526–38. 10.1104/pp.110.157370 PMC2923909 20566705

[48] Reichert A, He XZ, Dixon R. Phenylalanine ammonia-lyase (pal) from tobacco (nicotiana tabacum): characterization of the four tobacco pal genes and active heterotetrameric enzymes. Biochem J, 424(2), 233–42. 10.1042/BJ20090620 19725811

[49] Pant S, Huang Y. genes in sorghum and their responses to aphid infestation[J].Scientific Reports. 10.1038/s41598-022-25214-1 PMC9800386 36581623

[50] Hamberger B (2007). Genome-wide analyses of phenylpropanoid-related genes in Populus trichocarpa, Arabidopsis thaliana, and Oryza sativa: the Populus lignin toolbox and conservation and diversification of angiosperm gene families. Can J Bot.

[51] Shang QM, Li L, Dong CJ (2012). Multiple tandem duplication of the phenylalanine ammonia-lyase genes in Cucumis sativus L. Planta.

[52] Wu D-G, Yu ZHANQ (2020). Genome-wide identification and analysis of maize pal gene family and its expression profile in response to high-temperature stress. Pak J Bot.

[53] Rasool F, Uzair M, Naeem MK (2021). Phenylalanine Ammonia-lyase (PAL) genes family in wheat (Triticum aestivum L). Genome-Wide Charact Expression Profiling.

[54] Clark JW, Donoghue PCJ (2018). Whole-genome duplication and plant macroevolution. Trends Plant Sci.

[55] Fukasawa-Akada T, Kung SD, Watson JC (1996). Phenylalanine ammonia-lyase gene structure, expression, and evolution in Nicotiana. Plant Mol Biol.

[56] LI Q E, QIN Y, ZHENG Q M (2022). Codon bias and evolution analysis of phenylalanine ammonia-lyase gene [J]. J Biol.

[57] Rongrong L, Shaohua X,,Jialin L et al. Expression profile of a PAL gene from Astragalus membranaceus var. Mongholicus and its crucial role in flux into flavonoid biosynthesis.[J].Plant cell reports,2006,25(7):705–10. 10.1007/s00299-005-0072-7 16456646

[58] Pellegrini L, Rohfritsch O, Fritig B (1994). Phenylalanine ammonia-lyase in tobacco: molecular cloning and gene expression during the hypersensitive reaction to tobacco mosaic virus and the response to a fungal elicitor [. J] Plant Physiol.

[59] Cochrane FC, Davin LB, Lewis NG (2004). The Arabidopsis phenylalanine ammonia lyase gene family: kinetic characterization of the four PAL isoforms. Phytochemistry.

