# Supplementary material for: Identification and characterization of PAL genes involved in the regulation of stem development in Saccharum spontaneum L
Source: BMC Genom Data. 2024 Apr 30;25:38. doi: 10.1186/s12863-024-01219-9 (PMC11061975; doi:10.1186/s12863-024-01219-9)
Supplement: Supplementary file 1 — Supplementary Material 1 [file 12863_2024_1219_MOESM1_ESM.docx]

**Table S1.** qRT-PCR primers for *S. spontaneum* *PAL* gene.

| Name of primer | Primer sequences（5'→3'） |
| --- | --- |
| *SsPAL*11-F | AGGTCAACTCCGTCAACGAC |
| *SsPAL*11-R | CATGAGCTTGCCGATGTTGG |
| *SsPAL*15b-F | CTCATGTTCGCGCAGTTCTC |
| *SsPAL*15b-R | CTCTGGACGTGGTTGGTGAT |
| *SsPAL*15c-F | AACTCGGTCAACGACAACCC |
| *SsPAL*15c-R | GAGAACTGCGCGAACATGAG |
| 25s-F | ATAACCGCATCAGGTCTCCAA |
| 25s-R | CCTCAGAGCCAATCCTTTTCC |

**Table S2.** Basic information on the genes and encoded proteins of sugarcane cultivar R570 and sorghum *PAL* family.

| Sequence ID | NA | MW | *pI* | Instability Index | Aliphatic Index | Grand Average of Hydropathicity |
| --- | --- | --- | --- | --- | --- | --- |
| *SsPAL*1 | 659 | 70773.68 | 5.74 | 29.65 | 89.51 | -0.05 |
| *SsPAL*2 | 699 | 75347.12 | 5.69 | 34.8 | 92.33 | -0.086 |
| *SsPAL*3 | 694 | 74972.59 | 6.11 | 30.77 | 90.19 | -0.1 |
| *SsPAL*4a | 712 | 76833.67 | 6.18 | 31.55 | 93.62 | -0.115 |
| *SsPAL*4b | 703 | 75799.62 | 6.17 | 28.78 | 95.23 | -0.079 |
| *SsPAL*4c | 703 | 75853.66 | 6.17 | 29.01 | 93.71 | -0.096 |
| *SsPAL*4d | 703 | 75793.54 | 6.17 | 29.68 | 93.98 | -0.093 |
| *SsPAL*5a | 1054 | 114026.09 | 9.64 | 41.47 | 82.93 | -0.34 |
| *SsPAL*5b | 710 | 76182.8 | 5.74 | 29.17 | 88.99 | -0.1 |
| *SsPAL*5c | 714 | 76737.55 | 5.8 | 30.75 | 90.27 | -0.081 |
| *SsPAL*6 | 714 | 76910.98 | 5.89 | 34.09 | 92.72 | -0.088 |
| *SsPAL*7 | 716 | 77465.54 | 5.97 | 33.58 | 91.62 | -0.122 |
| *SsPAL*8 | 720 | 77779.83 | 5.97 | 33.96 | 90.85 | -0.126 |
| *SsPAL*9 | 894 | 96746.43 | 6.14 | 36.48 | 89.35 | -0.103 |
| *SsPAL*10 | 766 | 82077.54 | 5.77 | 35.06 | 89.74 | -0.087 |
| *SsPAL*11 | 713 | 76739.78 | 5.89 | 34.33 | 92.85 | -0.082 |
| *SsPAL*12 | 714 | 77001.06 | 5.89 | 35.07 | 92.44 | -0.094 |
| *SsPAL*13 | 714 | 76941.01 | 5.89 | 34.66 | 92.72 | -0.088 |
| *SsPAL*14 | 713 | 76753.85 | 5.7 | 34.78 | 92.85 | -0.082 |
| *SsPAL*15a | 707 | 76257.25 | 5.82 | 34.24 | 93.08 | -0.083 |
| *SsPAL*15b | 714 | 76984.04 | 5.97 | 34.93 | 92.17 | -0.1 |
| *SsPAL*15c | 698 | 75210.01 | 5.89 | 35.23 | 92.88 | -0.095 |
| *SsPAL*15d | 659 | 71580.63 | 7.56 | 38.42 | 86.24 | -0.227 |
| *SsPAL*16 | 1391 | 149933.3 | 6.07 | 34.3 | 91.59 | -0.106 |
| *SsPAL*17 | 714 | 77002.99 | 5.82 | 34.8 | 91.75 | -0.105 |
| *SsPAL*18 | 723 | 77773.6 | 5.94 | 31.71 | 94.33 | -0.118 |
| *SbPAL*1 | 771 | 83115 | 6.26 | 37.42 | 89.64 | -0.02 |
| *SbPAL*2 | 720 | 77870.92 | 5.97 | 35.29 | 90.18 | -0.072 |
| *SbPAL*3 | 720 | 77870.92 | 5.97 | 34.57 | 90.18 | -0.076 |
| *SbPAL*4 | 713 | 77599.72 | 5.89 | 34.57 | 93.25 | -0.079 |
| *SbPAL*5 | 714 | 76930.03 | 5.93 | 34.39 | 92.72 | -0.079 |
| *SbPAL*6 | 703 | 76823.91 | 6.17 | 33.79 | 93.71 | -0.08 |
| *SbPAL*7 | 704 | 76823.91 | 6 | 33.52 | 95.48 | -0.08 |
| *SbPAL*8 | 704 | 76809.88 | 6 | 33.52 | 95.48 | -0.08 |
| *ShPAL*1 | 709 | 76770.84 | 5.6 | 32.69 | 94.32 | -0.083 |
| *ShPAL*2 | 704 | 76602.32 | 6 | 32.69 | 95.63 | -0.089 |
| *ShPAL*3 | 718 | 76241.11 | 6.04 | 31.94 | 92.19 | -0.092 |
| *ShPAL*4 | 714 | 75809.6 | 6.05 | 31.81 | 92.44 | -0.096 |
| *ShPAL*5 | 714 | 75698.52 | 6.05 | 31.63 | 92.44 | -0.098 |
| *ShPAL*6 | 714 | 75630.34 | 6.04 | 29.4 | 92.44 | -0.119 |
| *ShPAL*7 | 703 | 75630.34 | 6.25 | 29.11 | 93.44 | -0.138 |
| *ShPAL*8 | 714 | 75602.33 | 5.8 | 29.1 | 88.21 | -0.138 |

**Table S3.** Collinear gene pairs in the *SsPAL* gene family.

| Collinear gene pairs | | | | | | | | | |
| --- | --- | --- | --- | --- | --- | --- | --- | --- | --- |
| Gene 1 | | | | | Gene 2 | | | | |
| Name | Gene ID | Chr | Start | End | Name | Gene ID | Chr | Start | End |
| *SsPAL3* | *Sspon.04G0008040-1T* | Chr4A | 22730416 | 22732560 | *SsPAL4b* | *Sspon.04G0008040-2B* | Chr4B | 19249974 | 19252115 |
| *SsPAL6* | *Sspon.04G0008040-1P* | Chr4A | 22918094 | 22920238 | *SsPAL8* | *Sspon.04G0024420-1B* | Chr4B | 19266689 | 19268741 |
| *SsPAL2* | *Sspon.04G0008040-2P* | Chr4A | 22666365 | 22668508 | *SsPAL8* | *Sspon.04G0024420-1B* | Chr4B | 19266689 | 19268741 |
| *SsPAL5a* | *Sspon.04G0008060-1A* | Chr4A | 22906780 | 22912376 | *SsPAL5b* | *Sspon.04G0008060-2B* | Chr4B | 19273240 | 19277397 |
| *SsPAL2* | *Sspon.04G0008040-2P* | Chr4A | 22666365 | 22668508 | *SsPAL4c* | *Sspon.04G0008040-3C* | Chr4C | 22787503 | 22789647 |
| *SsPAL5a* | *Sspon.04G0008060-1A* | Chr4A | 22906780 | 22912376 | *SsPAL9* | *Sspon.04G0032220-1C* | Chr4C | 22823601 | 22834706 |
| *SsPAL2* | *Sspon.04G0008040-2P* | Chr4A | 22666365 | 22668508 | *SsPAL13* | *Sspon.04G0008040-8P* | Chr4D | 23826676 | 23828819 |
| *SsPAL6* | *Sspon.04G0008040-1P* | Chr4A | 22918094 | 22920238 | *SsPAL4d* | *Sspon.04G0008040-4D* | Chr4D | 23900209 | 23902353 |
| *SsPAL6* | *Sspon.04G0008040-1P* | Chr4A | 22918094 | 22920238 | *SsPAL14* | *Sspon.04G0008040-4P* | Chr5A | 21988463 | 21990689 |
| *SsPAL4b* | *Sspon.04G0008040-2B* | Chr4B | 19249974 | 19252115 | *SsPAL4c* | *Sspon.04G0008040-3C* | Chr4C | 22787503 | 22789647 |
| *SsPAL8* | *Sspon.04G0024420-1B* | Chr4B | 19266689 | 19268741 | *SsPAL10* | *Sspon.04G0008040-6P* | Chr4C | 22841945 | 22844089 |
| *SsPAL5b* | *Sspon.04G0008060-2B* | Chr4B | 19273240 | 19277397 | *SsPAL9* | *Sspon.04G0032220-1C* | Chr4C | 22823601 | 22834706 |
| *SsPAL4b* | *Sspon.04G0008040-2B* | Chr4B | 19249974 | 19252115 | *SsPAL12* | *Sspon.04G0008040-11P* | Chr4D | 23788711 | 23795981 |
| *SsPAL8* | *Sspon.04G0024420-1B* | Chr4B | 19266689 | 19268741 | *SsPAL13* | *Sspon.04G0008040-8P* | Chr4D | 23826676 | 23828819 |
| *SsPAL8* | *Sspon.04G0024420-1B* | Chr4B | 19266689 | 19268741 | *SsPAL16* | *Sspon.04G0008040-5P* | Chr5B | 15823409 | 15829725 |
| *SsPAL10* | *Sspon.04G0008040-6P* | Chr4C | 22841945 | 22844089 | *SsPAL4d* | *Sspon.04G0008040-4D* | Chr4D | 23900209 | 23902353 |
| *SsPAL4c* | *Sspon.04G0008040-3C* | Chr4C | 22787503 | 22789647 | *SsPAL12* | *Sspon.04G0008040-11P* | Chr4D | 23788711 | 23795981 |
| *SsPAL10* | *Sspon.04G0008040-6P* | Chr4C | 22841945 | 22844089 | *SsPAL14* | *Sspon.04G0008040-4P* | Chr5A | 21988463 | 21990689 |
| *SsPAL11* | *Sspon.04G0008070-3C* | Chr4C | 22973069 | 22987554 | *SsPAL15a* | *Sspon.05G0007010-1A* | Chr5A | 22015355 | 22018342 |
| *SsPAL11* | *Sspon.04G0008070-3C* | Chr4C | 22973069 | 22987554 | *SsPAL15b* | *Sspon.05G0007010-2B* | Chr5B | 15842180 | 15844868 |
| *SsPAL10* | *Sspon.04G0008040-6P* | Chr4C | 22841945 | 22844089 | *SsPAL17* | *Sspon.04G0008040-7P* | Chr5C | 13199547 | 13201877 |
| *SsPAL11* | *Sspon.04G0008070-3C* | Chr4C | 22973069 | 22987554 | *SsPAL15c* | *Sspon.05G0007010-3C* | Chr5C | 13220742 | 13223184 |
| *SsPAL11* | *Sspon.04G0008070-3C* | Chr4C | 22973069 | 22987554 | *SsPAL15d* | *Sspon.05G0007010-4D* | Chr5D | 23089612 | 23092054 |
| *SsPAL14* | *Sspon.04G0008040-4P* | Chr5A | 21988463 | 21990689 | *SsPAL16* | *Sspon.04G0008040-5P* | Chr5B | 15823409 | 15829725 |
| *SsPAL15a* | *Sspon.05G0007010-1A* | Chr5A | 22015355 | 22018342 | *SsPAL15b* | *Sspon.05G0007010-2B* | Chr5B | 15842180 | 15844868 |
| *SsPAL14* | *Sspon.04G0008040-4P* | Chr5A | 21988463 | 21990689 | *SsPAL17* | *Sspon.04G0008040-7P* | Chr5C | 13199547 | 13201877 |
| *SsPAL15a* | *Sspon.05G0007010-1A* | Chr5A | 22015355 | 22018342 | *SsPAL15c* | *Sspon.04G0008040-7P* | Chr5C | 13220742 | 13223184 |
| *SsPAL15a* | *Sspon.05G0007010-1A* | Chr5A | 22015355 | 22018342 | *SsPAL15d* | *Sspon.04G0008040-7P* | Chr5D | 23089612 | 23092054 |
| *SsPAL16* | *Sspon.04G0008040-5P* | Chr5B | 15823409 | 15829725 | *SsPAL17* | *Sspon.04G0008040-7P* | Chr5C | 13199547 | 13201877 |
| *SsPAL15b* | *Sspon.05G0007010-2B* | Chr5B | 15842180 | 15844868 | *SsPAL15c* | *Sspon.04G0008040-7P* | Chr5C | 13220742 | 13223184 |
| *SsPAL16* | *Sspon.04G0008040-5P* | Chr5B | 15823409 | 15829725 | *SsPAL18* | *Sspon.04G0008040-10P* | Chr5D | 23077010 | 23080449 |
| *SsPAL15b* | *Sspon.05G0007010-2B* | Chr5B | 15842180 | 15844868 | *SsPAL15d* | *Sspon.04G0008040-7P* | Chr5D | 23089612 | 23092054 |
| *SsPAL17* | *Sspon.04G0008040-7P* | Chr5C | 13199547 | 13201877 | *SsPAL18* | *Sspon.04G0008040-10P* | Chr5D | 23077010 | 23080449 |
| *SsPAL15c* | *Sspon.05G0007010-3C* | Chr5C | 13220742 | 13223184 | *SsPAL15d* | *Sspon.04G0008040-7P* | Chr5D | 23089612 | 23092054 |
| alleles | | | | | | | | | |
| Gene 1 | | | | | Gene 2 | | | | |
| Name | Gene ID | Chr | Start | End | Name | Gene ID | Chr | Start | End |
| *SsPAL3* | *Sspon.04G0008040-1T* | Chr4A | 22730416 | 22732560 | *SsPAL4b* | *Sspon.04G0008040-2B* | Chr4B | 19249974 | 19252115 |
| *SsPAL6* | *Sspon.04G0008040-1P* | Chr4A | 22918094 | 22920238 | *SsPAL8* | *Sspon.04G0024420-1B* | Chr4B | 19266689 | 19268741 |
| *SsPAL2* | *Sspon.04G0008040-2P* | Chr4A | 22666365 | 22668508 | *SsPAL8* | *Sspon.04G0024420-1B* | Chr4B | 19266689 | 19268741 |
| *SsPAL5a* | *Sspon.04G0008060-1A* | Chr4A | 22906780 | 22912376 | *SsPAL5b* | *Sspon.04G0008060-2B* | Chr4B | 19273240 | 19277397 |
| *SsPAL2* | *Sspon.04G0008040-2P* | Chr4A | 22666365 | 22668508 | *SsPAL4c* | *Sspon.04G0008040-3C* | Chr4C | 22787503 | 22789647 |
| *SsPAL5a* | *Sspon.04G0008060-1A* | Chr4A | 22906780 | 22912376 | *SsPAL9* | *Sspon.04G0032220-1C* | Chr4C | 22823601 | 22834706 |
| *SsPAL2* | *Sspon.04G0008040-2P* | Chr4A | 22666365 | 22668508 | *SsPAL13* | *Sspon.04G0008040-8P* | Chr4D | 23826676 | 23828819 |
| *SsPAL6* | *Sspon.04G0008040-1P* | Chr4A | 22918094 | 22920238 | *SsPAL4d* | *Sspon.04G0008040-4D* | Chr4D | 23900209 | 23902353 |
| *SsPAL4b* | *Sspon.04G0008040-2B* | Chr4B | 19249974 | 19252115 | *SsPAL4c* | *Sspon.04G0008040-3C* | Chr4C | 22787503 | 22789647 |
| *SsPAL8* | *Sspon.04G0024420-1B* | Chr4B | 19266689 | 19268741 | *SsPAL10* | *Sspon.04G0008040-6P* | Chr4C | 22841945 | 22844089 |
| *SsPAL5b* | *Sspon.04G0008060-2B* | Chr4B | 19273240 | 19277397 | *SsPAL9* | *Sspon.04G0032220-1C* | Chr4C | 22823601 | 22834706 |
| *SsPAL4b* | *Sspon.04G0008040-2B* | Chr4B | 19249974 | 19252115 | *SsPAL12* | *Sspon.04G0008040-11P* | Chr4D | 23788711 | 23795981 |
| *SsPAL8* | *Sspon.04G0024420-1B* | Chr4B | 19266689 | 19268741 | *SsPAL13* | *Sspon.04G0008040-8P* | Chr4D | 23826676 | 23828819 |
| *SsPAL10* | *Sspon.04G0008040-6P* | Chr4C | 22841945 | 22844089 | *SsPAL4d* | *Sspon.04G0008040-4D* | Chr4D | 23900209 | 23902353 |
| *SsPAL4c* | *Sspon.04G0008040-3C* | Chr4C | 22787503 | 22789647 | *SsPAL12* | *Sspon.04G0008040-11P* | Chr4D | 23788711 | 23795981 |
| *SsPAL14* | *Sspon.04G0008040-4P* | Chr5A | 21988463 | 21990689 | *SsPAL16* | *Sspon.04G0008040-5P* | Chr5B | 15823409 | 15829725 |
| *SsPAL15a* | *Sspon.05G0007010-1A* | Chr5A | 22015355 | 22018342 | *SsPAL15b* | *Sspon.05G0007010-2B* | Chr5B | 15842180 | 15844868 |
| *SsPAL14* | *Sspon.04G0008040-4P* | Chr5A | 21988463 | 21990689 | *SsPAL17* | *Sspon.04G0008040-7P* | Chr5C | 13199547 | 13201877 |
| *SsPAL15a* | *Sspon.05G0007010-1A* | Chr5A | 22015355 | 22018342 | *SsPAL15c* | *Sspon.04G0008040-7P* | Chr5C | 13220742 | 13223184 |
| *SsPAL15a* | *Sspon.05G0007010-1A* | Chr5A | 22015355 | 22018342 | *SsPAL15d* | *Sspon.04G0008040-7P* | Chr5D | 23089612 | 23092054 |
| *SsPAL16* | *Sspon.04G0008040-5P* | Chr5B | 15823409 | 15829725 | *SsPAL17* | *Sspon.04G0008040-7P* | Chr5C | 13199547 | 13201877 |
| *SsPAL15b* | *Sspon.05G0007010-2B* | Chr5B | 15842180 | 15844868 | *SsPAL15c* | *Sspon.04G0008040-7P* | Chr5C | 13220742 | 13223184 |
| *SsPAL16* | *Sspon.04G0008040-5P* | Chr5B | 15823409 | 15829725 | *SsPAL18* | *Sspon.04G0008040-10P* | Chr5D | 23077010 | 23080449 |
| *SsPAL15b* | *Sspon.05G0007010-2B* | Chr5B | 15842180 | 15844868 | *SsPAL15d* | *Sspon.04G0008040-7P* | Chr5D | 23089612 | 23092054 |
| *SsPAL17* | *Sspon.04G0008040-7P* | Chr5C | 13199547 | 13201877 | *SsPAL18* | *Sspon.04G0008040-10P* | Chr5D | 23077010 | 23080449 |
| *SsPAL15c* | *Sspon.05G0007010-3C* | Chr5C | 13220742 | 13223184 | *SsPAL15d* | *Sspon.04G0008040-7P* | Chr5D | 23089612 | 23092054 |
| non-alleles | | | | | | | | | |
| Gene 1 | | | | | Gene 2 | | | | |
| Name | Gene ID | Chr | Start | End | Name | Gene ID | Chr | Start | End |
| *SsPAL6* | *Sspon.04G0008040-1P* | Chr4A | 22918094 | 22920238 | *SsPAL14* | *Sspon.04G0008040-4P* | Chr5A | 21988463 | 21990689 |
| *SsPAL8* | *Sspon.04G0024420-1B* | Chr4B | 19266689 | 19268741 | *SsPAL16* | *Sspon.04G0008040-5P* | Chr5B | 15823409 | 15829725 |
| *SsPAL10* | *Sspon.04G0008040-6P* | Chr4C | 22841945 | 22844089 | *SsPAL14* | *Sspon.04G0008040-4P* | Chr5A | 21988463 | 21990689 |
| *SsPAL11* | *Sspon.04G0008070-3C* | Chr4C | 22973069 | 22987554 | *SsPAL15a* | *Sspon.05G0007010-1A* | Chr5A | 22015355 | 22018342 |
| *SsPAL11* | *Sspon.04G0008070-3C* | Chr4C | 22973069 | 22987554 | *SsPAL15b* | *Sspon.05G0007010-2B* | Chr5B | 15842180 | 15844868 |
| *SsPAL10* | *Sspon.04G0008040-6P* | Chr4C | 22841945 | 22844089 | *SsPAL17* | *Sspon.04G0008040-7P* | Chr5C | 13199547 | 13201877 |
| *SsPAL11* | *Sspon.04G0008070-3C* | Chr4C | 22973069 | 22987554 | *SsPAL15c* | *Sspon.05G0007010-3C* | Chr5C | 13220742 | 13223184 |
| *SsPAL11* | *Sspon.04G0008070-3C* | Chr4C | 22973069 | 22987554 | *SsPAL15d* | *Sspon.05G0007010-4D* | Chr5D | 23089612 | 23092054 |
